# Rare Cases of Hibernomas Associated With Bilateral Pheochromocytoma: A Report of Two Cases

**DOI:** 10.7759/cureus.36503

**Published:** 2023-03-22

**Authors:** Samia Bentaleb, Amal Essouabni, Hayat Aynaou, Houda Salhi, Hanan El Ouahabi

**Affiliations:** 1 Department of Endocrinology, Diabetology, Metabolic Diseases and Nutrition, Hassan II University Hospital, Fez, MAR

**Keywords:** adipocyt, histology, pheochromocytoma, adrenal, hibernoma

## Abstract

Hibernoma is an infrequent benign lipomatous tumor that shows differentiation to brown fat. It is a slowly growing tumor of variable consistency but often firmer than a classic lipoma, mobile, and rarely infiltrating. To date, there are only a few cases of adrenal hibernoma in the literature.

Herein, we report two cases. The first one is that of a 24-year-old female presenting with a bilateral adrenal incidentaloma discovered in the setting of abdominal pain whose histological study individualized a bilateral pheochromocytoma associated with a hibernoma. The second case is that of a young male of 23 years old. He was operated on in adolescence (age 14) for a left pheochromocytoma. The outcome was marked by the recurrence of the Menards triad (“headache, palpitations, and sweating”) and hypertension at the age of 23 years. It was due to the appearance of a contralateral right pheochromocytoma, whose histological study showed an association with a hibernoma, and the genetic study revealed Von Hippel-Lindau (VHL) disease.

## Introduction

Hibernoma is a rare benign adipocytic tumor (1.6%) that develops from brown adipose tissue. Described by Merckel in 1906, the term hibernoma (by Gery in 1914) refers to hibernating animals in which brown fat is playing a crucial role in energy and thermal regulation. Lipomatous adrenal tumors are unusual, accounting for 4.8% of all primary adrenal tumors. The most common ones are myelolipomas [1]. We report two cases of adrenal hibernomas discovered incidentally on a pathological study of a pheochromocytoma.

## Case presentation

First clinical case report

**Figure 1:**
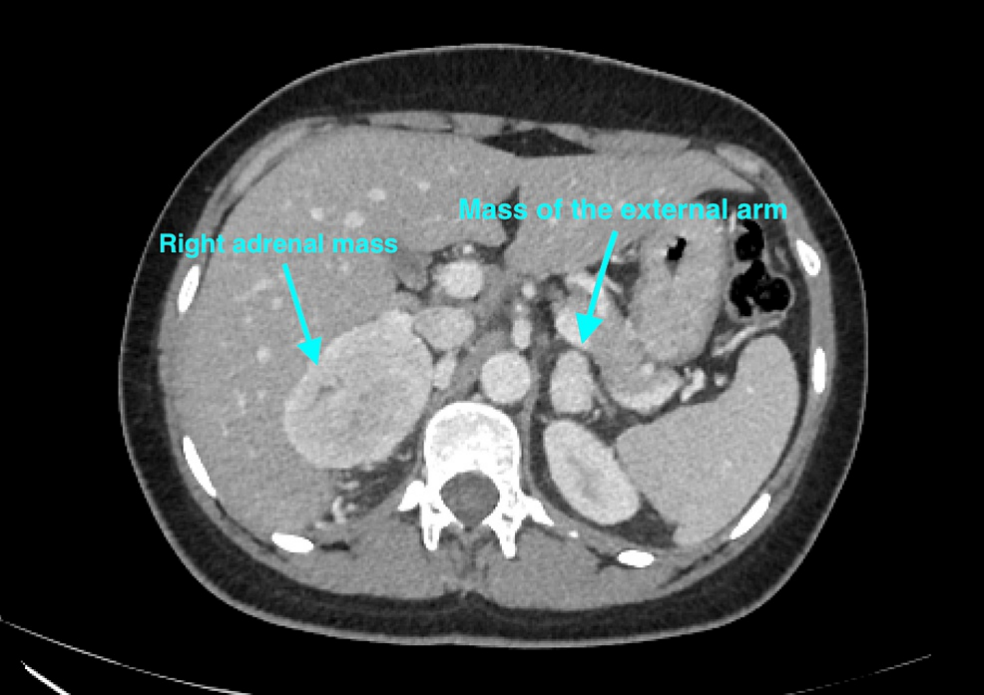
Cross section of an abdominal CT scan showing one right adrenal mass 55 × 70 × 50 mm in diameter and the other at the level of the medial arm of the left adrenal measuring 21 × 15 × 19 mm CT: computed tomography

A 24-year-old female, with an unremarkable medical history, was referred for exploration of a bilateral adrenal incidentaloma. It was detected by an abdominal computed tomography (CT) scan performed in the setting of diffuse abdominal pain. The patient reported frontal headaches, paroxysmal sweats, and palpitations in relation to the Menards triad, as well as a 7% weight loss over two months. On adrenal CT, the right mass was hypodense, with regular contours, without individualization of microcalcifications, with an estimated absolute washout of 76% and a relative washout of 42%, and the other mass at the level of the medial arm of the left adrenal gland (Figure 1).

**Figure 2:**
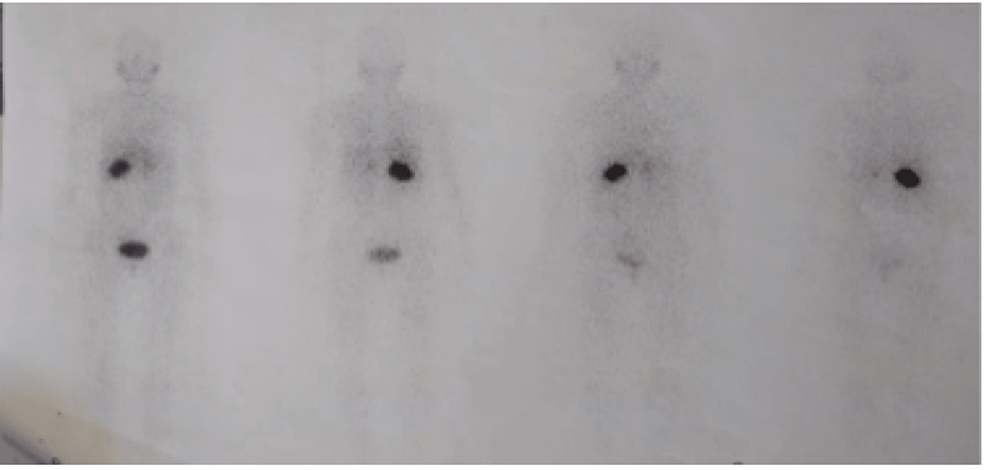
MIBG scintigraphy showing fixation opposite both adrenals MIBG: meta-iodobenzylguanidine

The secretory assessment showed positive urinary methoxylates (normetadrenaline at 16 × normal). Meta-iodobenzylguanidine (MIBG) scintigraphy showed a clear fixation defect in front of the two adrenal areas, corresponding to the pheochromocytomas described on the diagnostic CT scan, and an absence of secondary distant localizations (Figure 2).

The genetic study for Von Hippel-Lindau (*VHL*) gene was negative, and that for the rearranged during transfection (*RET*) gene is in progress.

After being prepared with an alpha blocker, the patient underwent bilateral laparoscopic adrenalectomy. The histological study showed a tumor proliferation arranged in nests and cords with abundant vascularization. It was made of medium-sized cells with a round vesicular nucleus, focal hyperchromasia, absence of pleomorphism, and mitoses estimated at 5/10 high-power field, leading to the diagnosis of a bilateral pheochromocytoma (Pheochromocytoma of the Adrenal gland Scaled Score (PASS) of 3). It was associated with a benign adipocyte proliferation with multivacuolar cytoplasm suggestive of a hibernoma. The postoperative outcome was favorable; the patient was discharged after 10 days with adequate hydrocortisone replacement.

Second clinical case report

A 23-year-old male, with a familial history of pheochromocytoma and VHL disease (a genetic study in a cousin revealed a heterozygous family variation in exon 1 of the *VHL* gene), was operated on at the age of 14 for a left pheochromocytoma with no follow-up.

The patient was seen in the clinic for grade III hypertension associated with a positive Menards triad and an undetermined weight loss.

The secretory assessment showed positive urinary methoxylates (normetanephrine more than 24 times the upper limit of normal, and 3 ortho methyldopamine at 1.09 times the upper limit of normal).

**Figure 3:**
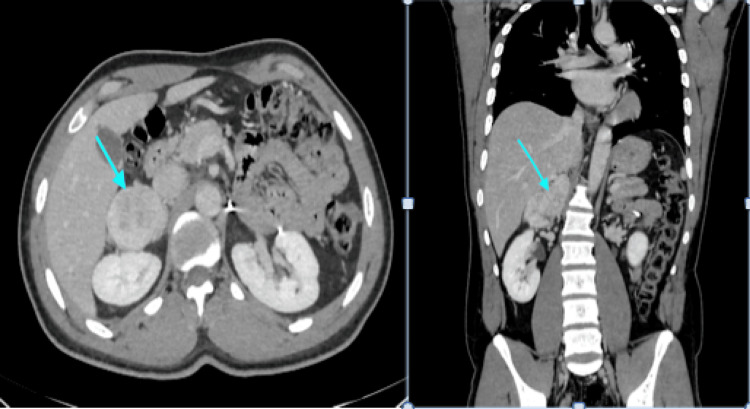
A coronal and transverse section of an abdominal CT scan showing a right adrenal tissue mass with lobulated contours, heterogeneously enhanced after contrast injection, measuring 76 × 54 × 34 mm in diameter CT: computed tomography

Adrenal CT showed a well-limited, poly-lobed, hypervascular, intensely and heterogeneously enhanced right adrenal tissue mass at the arterial and portal times, measuring 40 × 60 × 88 mm in diameter, interpreted as a hypervascularized right adrenal tumor process, in relation with his pheochromocytoma (Figure 3). An adrenal MRI performed in search of an extension to the surrounding organs showed a lobulated right adrenal lodge tissue mass, described in T1 hypointense and T2 hyperintense, diffusion-restrictive, progressively and heterogeneously enhanced after the injection of contrast medium, measuring 76 × 54 × 34 mm in diameter.

**Figure 4:**
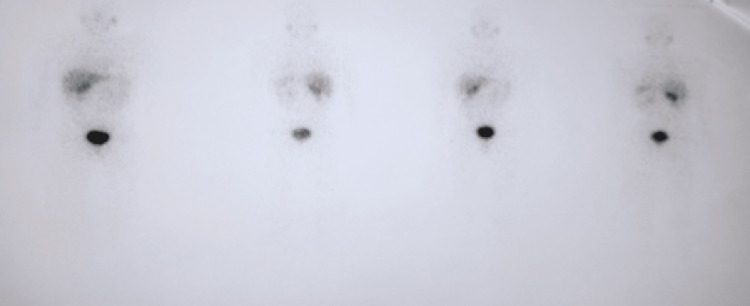
Scintigraphic image in favor of a focus on intense MIBG uptake opposite the right adrenal cavity, suggesting a pheochromocytoma MIBG: meta-iodobenzylguanidine

As part of the extension assessment, MIBG scintigraphy was performed and showed a focus on intense uptake in the right adrenal gland, which evokes first a pheochromocytoma, without any other distant localizations (Figure 4).

 A genetic study was performed and came back in favor of VHL disease.

**Figure 5:**
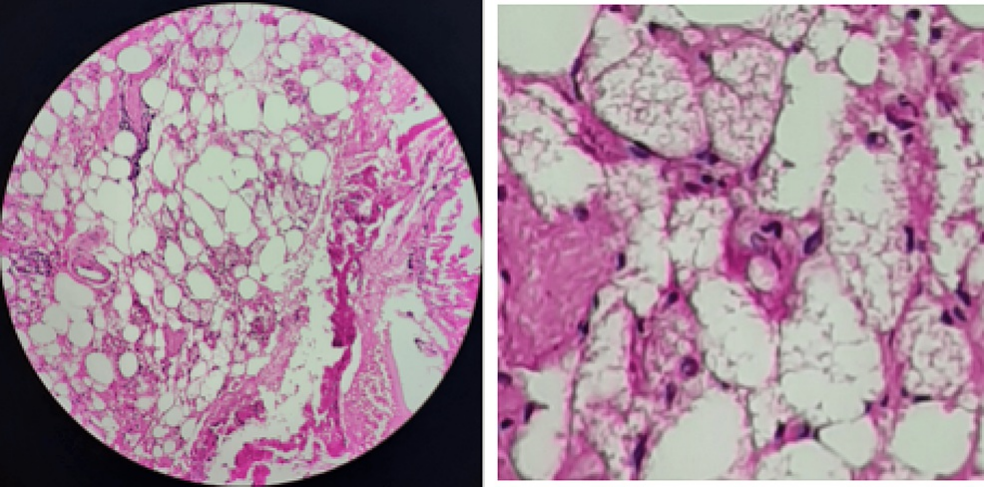
Image of the histopathological evaluation of the adrenal hibernoma in our patient sampling of the satellite mass of the pheochromocytoma showing a benign tumor proliferation made of brown adipocytes arranged in lobules; the cells are endowed with an abundant micro-vacuolar cytoplasm with a non-atypical round nucleus and without mitosis figure

After being prepared with an alpha blocker, the patient underwent a laparoscopic right adrenalectomy. The histological study showed a pheochromocytoma of nonaggressive potential (PASS of 3) associated with a hibernoma (Figure 5).

## Discussion

We described adrenal hibernomas discovered incidentally on histological evaluation of two patients with pheochromocytoma.

Hibernoma is a rare benign lipomatous tumor. It could be subcutaneous or intramuscular, and its cells show a differentiation to brown fat [1]. In adults, the brown fat is reduced to a remnant state, and remnants of this fat are found in the scapular region, the neck around the large vessels, the kidney, and the heart [2]. Adrenal localization is rare; up to now, a few cases of adrenal hibernoma have been reported. One of those was described by Schwartz and Wasson; it was of a 42-year-old female with a 30-mm solid right adrenal mass that was increasing in volume and was biologically inactive. The histological study revealed a myelolipoma measuring 33 mm in association with an 11-mm hibernoma [3]. Another case was described by Val-Bernal et al. in a 55-year-old female; it was an incidentally discovered mass of 26 mm associated with Conn’s syndrome. The hibernoma measured 17 mm and consisted of univacuolated mature adipose cells with central or paracentral nuclei [4]. Adrenal hibernoma combined with a pheochromocytoma was found in our patients at a younger age as their tumors were discovered incidentally in the operating room.

Clinically, it is a tumor of variable consistency but often firmer than a lipoma, mobile, rarely infiltrative, and slow-growing. The associated symptoms include pain related to the compression of surrounding structures and rarely weight loss in relation to the excessive metabolic activity of the tumor tissue [5]. In our patients, we note the presence of the Menards triad with weight loss, which is due to the pheochromocytoma, without other symptoms.

Radiological examinations (CT scan and magnetic resonance imaging (MRI)) orient the diagnosis of hibernoma and especially rule out the differential diagnosis of liposarcoma [6]. However, the diagnosis confirmation remains histological [7,8]. CT shows a well-limited tumor of intermediate density between muscle and fat and devoid of calcification, which is enhanced by injection of contrast medium. On MRI, the hibernoma corresponds to a T1 hyperintense and T2 hyperintense mass with inhomogeneous patches of hypointense within the tumor. In addition, the density is enhanced with gadolinium injection [3,6]. In our two patients, adrenal CT and MRI showed just the mass in relation to pheochromocytoma without individualization of the hibernoma.

Macroscopically, this tumor is brownish-yellow, well-encapsulated, and highly vascularized, roughly resembling a lipoma. On microscopic examination, the tumor contains a mixture of cells of different degrees of differentiation. There is an amalgam of mature adipocytes, multivacuole cells, and round cells with central nuclei that correspond to “brown adipocytes” [3,8]. In our patients, the histological study showed a hibernoma measuring 3 cm in the first case and was composed mainly of adipocytes with macro-multivacuolar cytoplasm and showed several cytoplasmic granulations. They have a central round nucleus of small size. Sometimes, there are foci (>30%) made of adipocytes with vacuolated cytoplasm and peripheral nuclei. These cells do not show cytonuclear atypia or mitosis. This proliferation is arranged in lobules separated by fibrous septa and thin-walled capillary structures. In the second patient, it measured 2 × 2.5 cm, made of brown adipocytes arranged in lobules. The cells have an abundant micro-vacuolar cytoplasm with a round non-atypical nucleus and without mitosis figure. The curative treatment is essentially a complete surgical excision, allowing a histological confirmation of the diagnosis and avoiding any recurrence. Indeed, our two patients benefited from a surgical resection by laparoscopy with a good outcome. To our knowledge, no malignant transformation has been reported in the literature [4].

## Conclusions

Hibernoma is a rare benign adipocytic tumor, often unrecognized, affecting young patients with a slight female predominance. It should be suspected when a firm, richly vascularized, brownish-colored lipomatous tumor is found, which can be worrisome during surgical removal. Adrenal localization of the hibernoma remains rare. Imaging (especially MRI) orients the diagnosis and omits its differential diagnosis.
